# The extracts of *Astragalus membranaceus* enhance chemosensitivity and reduce tumor indoleamine 2, 3-dioxygenase expression

**DOI:** 10.7150/ijms.33106

**Published:** 2019-08-06

**Authors:** Naphichaya Phacharapiyangkul, Li-Hsien Wu, Wei-Ya Lee, Yi-Hsuan Kuo, Yueh-Jung Wu, Huei-Pu Liou, Yung-En Tsai, Che-Hsin Lee

**Affiliations:** 1 Department of Microbiology, Faculty of Pharmacy, Mahidol University, Bangkok, Thailand; 2 Department of Biological Sciences, National Sun Yat-sen University, Kaohsiung, Taiwan;; 3 Department of Surgery, Kaohsiung Armed Forces General Hospital, Kaohsiung, Taiwan;; 4 Division of Nephrology, Department of Internal Medicine, Kaohsiung Armed Forces General Hospital, Kaohsiung, Taiwan;; 5 Department of Medical Research, China Medical University Hospital, China Medical University, Taichung, Taiwan;; 6 Department of Medical Laboratory Science and Biotechnology, Kaohsiung Medical University, Kaohsiung, Taiwan.

**Keywords:** *Astragalus membranaceus*, PG2, Connexin 43, Indoleamine 2, 3-dioxygenase, Combination therapy

## Abstract

*Astragalus membranaceus* has been shown to possess anti-inflammation and antitumor properties. Several studies have indicated that extracts of* Astragalus membranaceus* (PG2) have growth inhibitory effects on tumor. However, the effect of PG2 on enhancing the chemotherapy, modulating tumor immune escape and their mechanism of action is unknown and need further investigation. Connexin (Cx) 43 is ubiquitous in cells and involved in facilitating the passage of chemotherapeutic drugs to bystander tumor cells. The indoleamine 2, 3-dioxygenase (IDO) depletes tryptophan, reduces the active T cell number and destroys immune surveillance. Herein, we provide evidence that the treatment of PG2 induced Cx43 expression, decreases IDO expression and enhances the distribution of chemotherapeutic drug. However, the effects of combination therapy (PG2 plus cisplatin) in animal models significantly retarded tumor growth and prolonged the survival. We believe that the information provided in this study may aid in the design of future therapy of PG2, suggest suitable combinations with chemotherapies.

## Introduction

*Astragalus membranaceus* is a traditional Chinese medicine and is used for promoting health. PG2 is a polysaccharide purified from the root of *Astragalus membranaceus* and has been used in cancer patients as a cancer-related fatigue agent 1. PG2 showed potential in immunomodulation, anti-oxidant and anti-inflammation activity 2-4. Although the growing evidences implied that PG2 exhibits potential antitumor activities 5, we still do not fully understand how the accurate mechanisms of its helpful response for the combination therapy of PG2 and cisplatin.

The gap junction is involved in tissue homeostasis and small molecule transference 6. Connexin (Cx) 43 is one of the wildly-studied connexin isoform and reduced in various tumors 7. Cx43 facilitates the passage of chemotherapeutic drugs between neighboring tumor cells and enhances the efficiency of chemotherapy 8, 9. The molecules-upregulated Cx43 expression in tumors might have some therapeutic advantages 10. Interesting evidence showed that with Cx43 expression comes chemotherapy-mediated response 11, 12.

PG2 not only regulated the growth of solid tumors but also host immunoresponses 13. Tumor cells could reduce the attack from immune cells by expressing indoleamine 2, 3-dioxygenase 1 (IDO) 7. IDO leads to effector T cell apoptosis through depletion of tryptophan and accumulation of kynurenine in tumor microenvironment 14. In this study, we demonstrated that the PG2 enhanced the chemotherapy by upregulating of Cx43 and stimulated host immunity by reducing the expression of IDO. The aim of the present study was to characterize the mechanism of action of PG2 on enhancing chemotherapy and overcoming tumor immunotolerance.

## Materials and Methods

### Reagents, cells and mouse

PG2 purified from the root of Astragalus membranaceus and purchased from PhytoHealth Corp (Taipei, Taiwan). The working concentrations of the various inhibitors were as follows: 25 μM SB203580 (Sigma-Aldrich, St. Louis, MO), 20 μM PD98059 (Sigma-Aldrich), or 10 μM SP600125 (Sigma-Aldrich) (Chang et al., 2013). Cells were pretreated various inhibitors for 2 h, then PG2 (100 ng/ml) was added to cells for 24 h. Cisplatin and 5-fluouracil (5-FU) were purchased from the Sigma-Aldrich. The A549 (human lung cancer cell), PC9 (human lung cancer cells), B16F10 (murine melanoma cells) and LL2 (murine lung carcinoma ) cells were cultured in Dulbecco's modified Eagle's medium containing 10% fetal bovine serum, 1% glutamine, and 1% Penicillin-Streptomycin (100 units/mL penicillin and 100 μg/mL streptomycin) at 37℃ in 5% CO2 condition. Jurkat cell line (human T lymphocyte) maintained in HyClone RPMI 1640 medium containing 10% fetal bovine serum. The C57BL/6 mice were purchased from the National Laboratory Animal Center of Taiwan. The experimental protocol was approved by the Laboratory Animal Care and Use Committee of the National Sun Yat-sen-University (permit number: 10635).

### Cell proliferation assay

Cells (10^5^/well) were treated with various concentration of cisplatin and PG2 in serum- free medium for 24 h. Cell proliferation was assessed by Cell Counting Kit-8 (Sigma-Aldrich) according to the manufacturer's instructions. The supernatants of PG2-treated B16F10 or LL2 were added to Jurkat cells mixed with equal amount of fresh medium. After 3 days, cell survival was assessed using the trypan blue exclusion assay 7.

### Western blot analysis

Cells with treatments were collected and lysed in lysed buffer (150 mM NaCl, 1% Nonidet P-40 (NP-40), 50 mM Tris-Cl). The protein content in each sample was determined by bicinchoninic acid (BCA) protein assay (Pierce Biotechnology, Rockford, IL, USA). Quantified each sample add 4 × SDS sample dye and then sample were denatured for 10 min at 95°C. Proteins were fractionated on SDS-PAGE, transferred onto Hybond enhanced chemiluminescence nitrocellulose membranes (Amersham, Little Chalfont, UK) and detected with antibodies against Cx43 (synthetic peptide corresponding to the C-terminal segment of the cytoplasmic domain of human/rat Cx43, C6219, Sigma-Aldrich), extracellular signal-regulated kinase (ERK) (Abcam, Cambridge, UK), phosphor-ERK(Abcam), p38 (Abcam), phosphor-p38 (Abcam), c-jun N terminal kinase (JNK) (Abcam), phosphor-JNK (Abcam), IDO (synthetic peptide between 79-105 amino acids from the central region of human IDO, PA5-24598, ThermoFisher Scientific, Waltham, MA), tryptamine 2, 3 dioxygenase (TDO) (MyBioSource Inc, San Diego, CA, USA ) and β-actin (Sigma-Aldrich). Rabbit anti-mouse IgG-peroxidase antibody (Sigma-Aldrich), donkey anti-goat IgG-peroxidase antibody (Santa Cruz Biotechnology, Santa Cruz, CA) and goat anti-rabbit IgG-peroxidase antibody (Sigma-Aldrich) were used as the secondary antibody and protein-antibody complexes were visualized by enhanced chemiluminescence system (T-Pro Biotechnology, New Taipei City, Taiwan).

### IDO functional assay

All cells incubation were at 37°C, 5% CO2. B16F10 cells were plated in 12 well waited for attachment, and incubated with either different concentrations of PG2 for 24 h. The medium of cells treated with PG2 would be collected. The supernatants were harvested with 30% TCA (Sigma-Aldrich) at 50°C, 30 min. The supernatants and TCA were mixed in an equal amount of Erchlich' s reagent (Sigma-Aldrich). The absorbance of kynurenine was detected by spectrophotometer at a wavelength of A490. The data were performed as percentage of control 7.

### Animal study

We used subcutaneous B16F10 and LL2 tumor models to evaluate the antitumor efficacy of combination treatment with PG2 and cisplatin. Groups of 7 mice that inoculated subcutaneously with B16F10 or LL2 cells (10^6^) at day 0 were intraperitoneal injected with PG2 (50 mg/kg) at day 7, day 10, day 13 followed by cisplatin (2 mg/kg) at days 8, 11, and 14, or with either treatment alone. The control mice were treated with PBS. All of the mice were monitored for tumor growth and survival as previously described 6.

### Immunohistochemistry assays

To analyze cell infiltrates in the tumors, groups of 3 mice that inoculated subcutaneously with B16F10 or LL2 cells (10^6^) at day 0 were intraperitoneal injected with PG2 (50 mg/kg) at day 7, day 10, day 13 followed by cisplatin (2 mg/kg) at days 8, 11, and 14, or with either treatment alone. The control mice were treated with PBS. The tumors were removed, washed with PBS, fixed in 3.7% formaldehyde, and embedded in paraffin ay day 15. Tumor tissues were then processed in 5-μm sections and stained with incubated with rat anti-mouse CD4 (GeneTeX, inc, Irvine, CA, USA), or mouse anti-mouse CD8 (GeneTex, Inc.) antibody. After sequential incubation with appropriate peroxidase-labeled secondary antibody and 3',3'-diaminobenzendine (DAB) as substrate chromogen, the slides were counterstained with hematoxylin.

### Statistical analysis

All data were expressed as mean ± standard deviation (SD). The unpaired, two-tailed Student's t test was used to determine differences between groups. Any P value less than 0.05 is regarded statistically significant.

## Results

### PG2 exerted a positive effect on Cx43 expression

**Figure 1:**
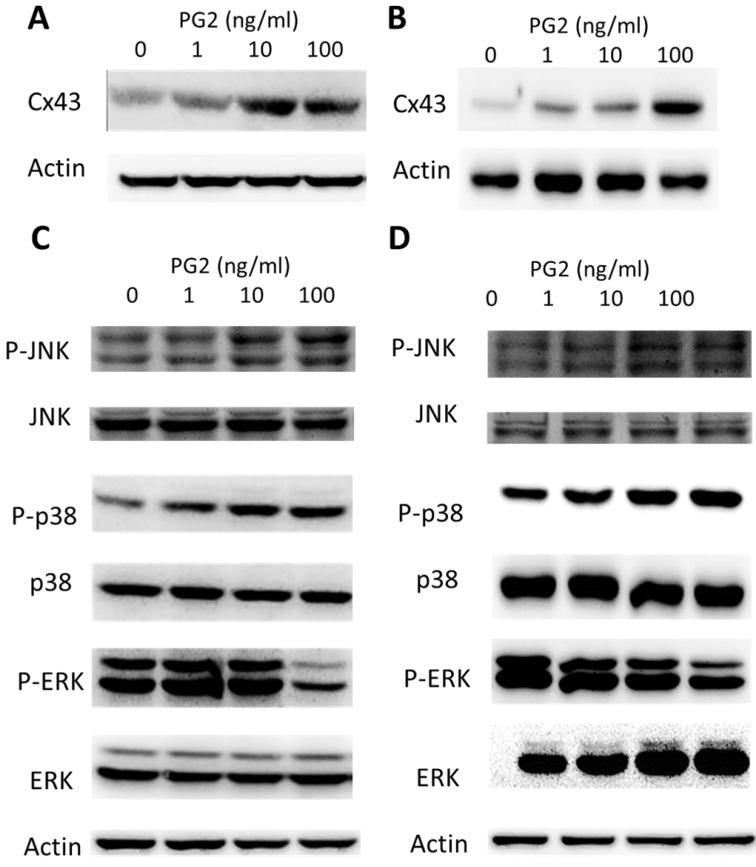
PG2-mediated Cx43 protein expression. PG2 induced Cx43 protein expression in (A) B16F10 and (B) LL2 cells in a dose-dependent manner. After treatment with PG2 (0-100 ng/ml) for 24 h, the expression of Cx43 levels in B16F10 and LL2 cells were measured by Western blotting. PG2 induced Cx43 expression through MAPK signal pathways. PG2 induced MAPK signaling pathways in (C) B16F10 and (D) LL2 cells in a dose-dependent manner. After treatment with PG2 (0-100 ng/ml) for 24 h, the expression of MAPK signaling pathways in B16F10 and LL2 cells were measured by Western blotting.

**Figure 2:**
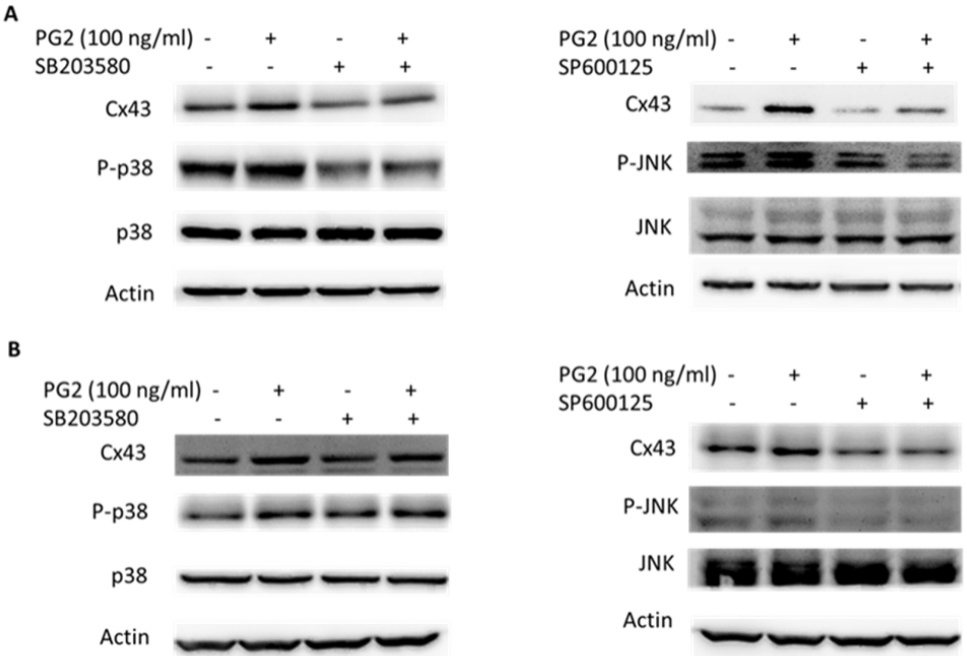
MAPK inhibitors reduced PG2-induced Cx43 expression. The tumor cells were treated with PG2 (100 ng/ml) for 24h. The cells were lysed and protein expression of Cx43, P38, P-P38, JNK, and P-JNK were measured. After treatment of cells with inhibitor for p38 (SB203580) and JNK (SP600125) for 1h, The B16F10 (A) and LL2 (B) cells were treated with PG2 (100 ng/ml) for 24h. The cells were lysed and protein expression of Cx43, P-P38, P38, JNK and P-JNK was examined by Western blotting.

To determine the effects of PG2 on the expression of Cx43 in B16F10 and LL2 cells, tumor cells were treated with different concentrations of PG2, and then analyzed by Western blotting. Tumor cells treated with PG2 at different concentrations showed increases of Cx43 expression compared to untreated cells in a dose dependent manner (Fig. 1 A and B). Tumor cells treated with PG2 (100 ng/ml) significantly increased the expression of Cx43. In the subsequent analyses, the concentration of PG2 (100 ng/ml) was used. The potential signaling pathways in PG2-induced Cx43 expression were examined in tumor cells. The mitogen-activated protein kinases (MAPK) signaling pathways have been demonstrated to involve in the expression of Cx43 6, 11,15. As shown in Fig. 1 C and D, the phosphorylation of c-jun N terminal kinase (JNK), and p38 were increased after PG2 treatment, but not the phosphorylation of extracellular signal-regulated kinase (ERK). Furthermore, the inhibitor of p38 (SB203580) and JNK (SP600125) reduced the PG2-induced Cx43 protein expression in B16F10 cells (Fig. 2A). The similar results were observed in LL2 cells (Fig. 2B). By using the inhibitor of p38 and JNK influenced PG2-induced Cx43 expression in tumor cells. These results suggest that MAPK signaling pathway might involve in the PG2-induced the expression of Cx43.

### PG2 enhanced cisplatin-induced cell death

**Figure 3:**
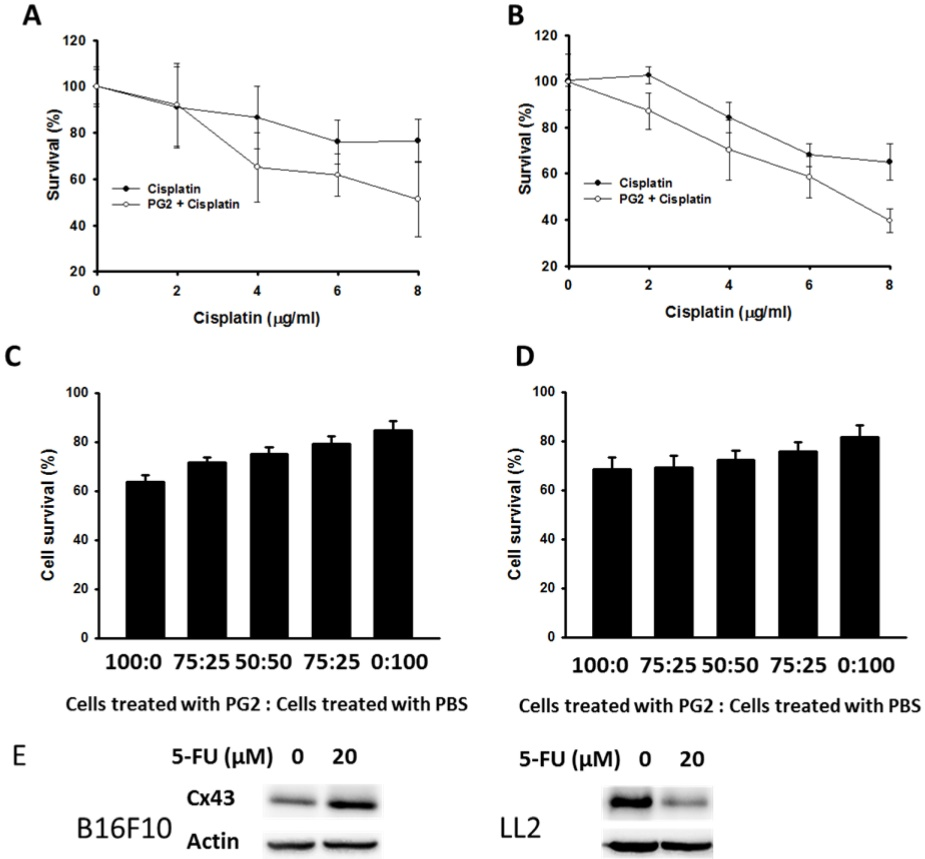
PG2 in combination with cisplatin exerted bystander cytotoxic effects. PG2-treated or control cells were exposed to cisplatin (0-8 μg/ml) for 48 h followed by determination of (A) B16F10 and (B) LL2 cell viability by the WST-1 assay. (n = 6, data are mean± SD). (P<0.001 for PG2 + Cisplatin versus Cisplatin). PG2-treated and PBS-treated cells were mixed in various ratios to generate 100, 75, 50, 25 and 0% PBS-treated cells. These (C) B16F10 and (D) LL2 cells were treated with cisplatin (4 μg/ml) for 48 h. Cell viability was determined by the WST-1 assay. (n = 6, data are mean± SD). (E) PG2-treated or control cells were exposed to 5-FU (0-20 nM) for 48 h. The cells were lysed and protein expression of Cx43, and IDO were measured.

PG2 could enhance the expression of Cx43 in B16F10 and LL2 cells. The upregulation of Cx43 comes the chemotherapeutic sensitivity of tumor cells. Cell viability was quantified by using a WST-1 assay. PG2 in combination with cisplatin significantly reduced cell proliferation compared with cell treated with cisplatin. The phenomenon was observed in two cell lines (B16F10 and LL2) (Fig. 3 A and B). To obtain more insight into the bystander effect of Cx43, by coculturing the PG2-treated cells with PBS-treated cells at varying ratios in the presence of cisplatin for 24 h, the cell viability was observed (Fig. 3 C and D) 11. Previously, cisplatin exits the inhibition of Cx43 expression. Hererin, we used another chemotherarutic drug (5-FU) to observe the effect of chemotherapeutic drug on Cx43 expression. The B16F10 cells treated with 5-FU enhanced Cx43 expression. Conversely, the phenomena was not observed in LL2 cells (Fig. 3E). The cisplatin (4μg/ml) slightly induced cytotoxicity in PBS-treated cells. The numbers of cell death were accompanied with the percentage of PG2-treated cells. The bystander effect was significantly observed in tumor cells treated with PG2 plus cisplatin.

### Inhibited IDO expression by PG2 treatment

**Figure 4:**
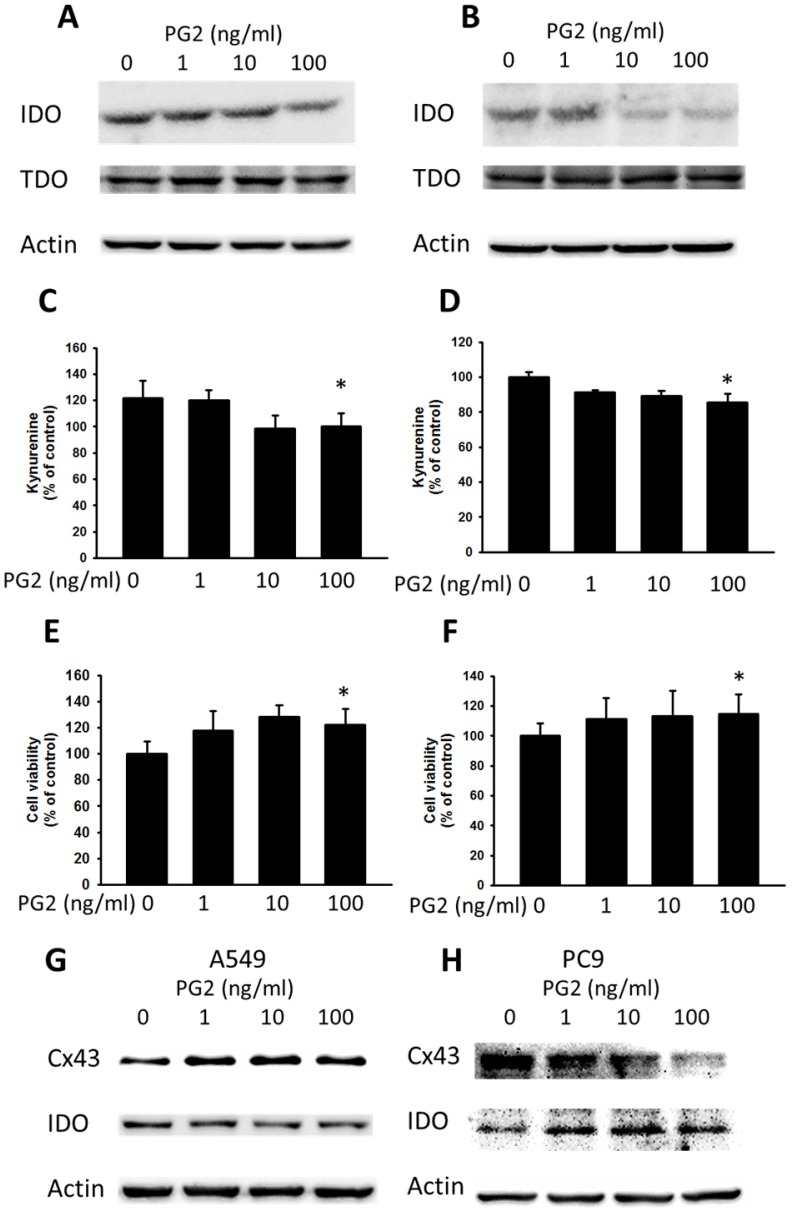
The effects of PG2 on the expression and function of IDO. PG2 inhibited IDO protein expression in (A) B16F10 and (B) LL2 cells in a dose-dependent manner. After treatment with PG2 (0-100 ng/ml) for 24 h, the expression of IDO and TDO levels in B16F10 and LL2 cells were measured by Western blotting. The kynurenine assay was used for the kynurenine production in (C) B16F10 and (D) LL2 cells. The conditioned medium of (E) B16F10 and (F) LL2 after treated with indicated concentrations of PG2 for 24 h mixed with an equal amount of original medium. T cells were cultured in media conditioned from tumor cells for 72 h. The cell number were measured by staining with trypan blue. (n = 6, data are mean± SD. * P < 0.05). PG2 induced Cx43 and inhibited IDO protein expression in (G) A549 and (H) PC9 cells in a dose-dependent manner. After treatment with PG2 (0-100 ng/ml) for 24 h, the expression of Cx43 and IDO levels in A549 and PC9 cells were measured by Western blotting.

Cx43 has introduced tumor suppressor to regulate various molecules 10. The downregulation of Cx43 has been shown to induce the expression of IDO in tumors 7. Enhanced Cx43 expression in tumor cells might result in decreased IDO expression and IDO-mediated function. Previously, our studies found that some molecules could enhance the expression of Cx43 and reduce the expression of IDO 7. The TDO is closely related to IDO and also involved in tryptophan metabolism. As shown in Fig. 4 A and B, PG2 possessed the ability of decreasing IDO expression in dose-dependent manner, but not TDO. To further confirm the result, the accumulation of kynurenine, IDO-mediated tryptophan metabolites, result in the inhibition of T cell proliferation. PG2 significantly reduced the production of kynurenine in two tumor cells (Fig. 4 B and D). Furthermore, the T cell viability was higher after the conditioned medium derived from the tumor cells treated with PG2 than that derived from the tumor cells treated with PBS (Fig. 4 E and F). Meanwhile, the human cancer cells (A549 and PC9) were observed the negative correlation of Cx43 and IDO expression after PG2 treatment (Fig. 4 G and H). The results suggested that PG2 inhibited the production and function of IDO in tumor cells.

### The combination therapy of PG2 and cisplatin* in vivo*

**Figure 5:**
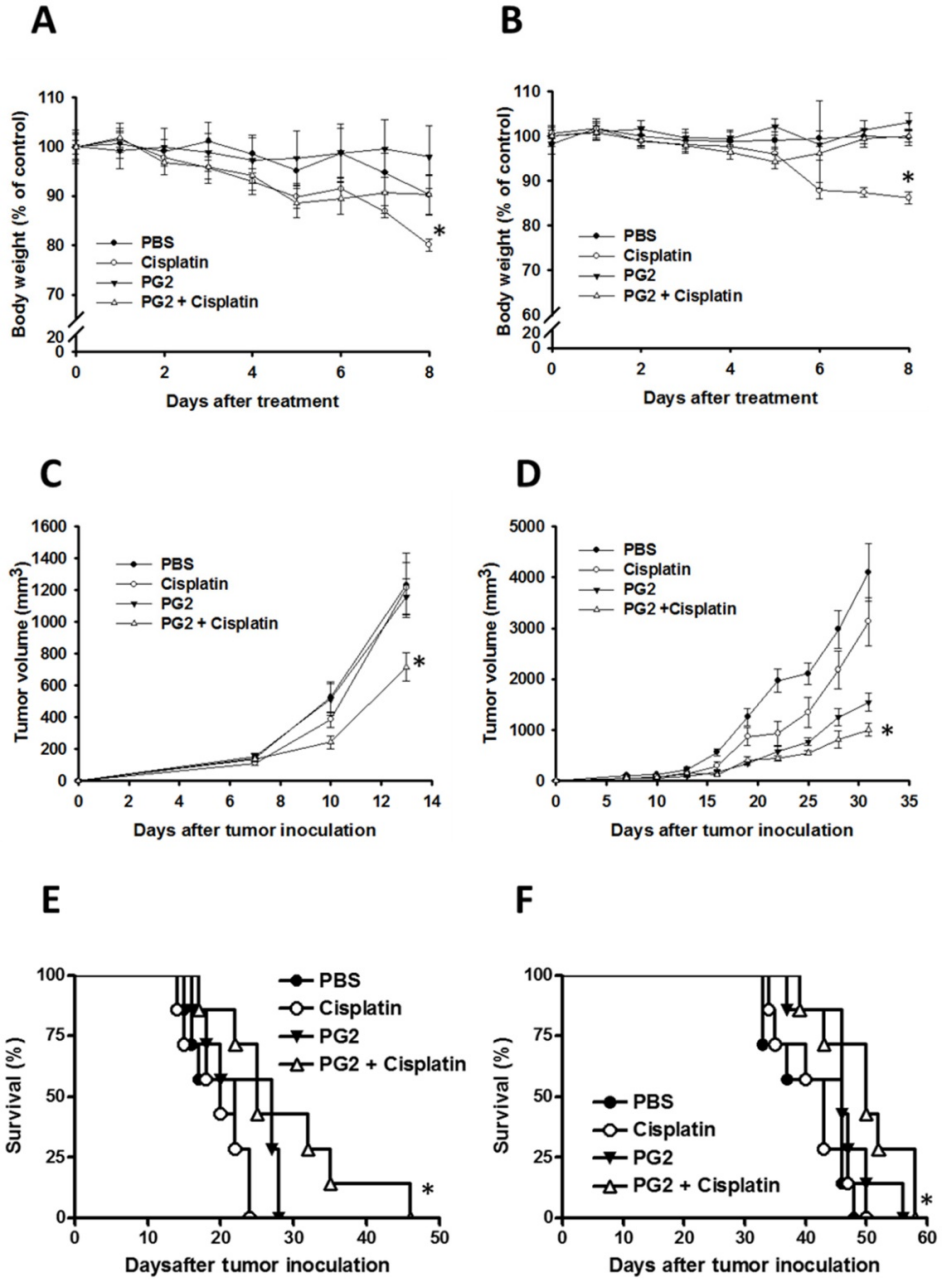
Additive antitumor effects of PG2 in combination with cisplatin on tumors. Mice that had been inoculated subcutaneously with B16F10 or LL2 cells (10^6^) at day 0 were intraperitoneal injected with PG2 (50 mg/kg) at day 7, day 10, day 13 followed by cisplatin (2 mg/kg) at days 8, 11, and 14, or with either treatment alone. The control mice were treated with PBS. The (A, B) body weight and (C, D) tumor volumes among different treatment groups were compared in mice bearing (A, C) B16F10 and (B, D) LL2 tumors. (mean ± SEM, n = 7, * P < 0.05). Kaplan-Meier survival curves of the mice bearing (E) B16F10 and (F) LL2 tumors (P<0.05 for PG2+cisplatin versus PG2, cisplatin, or PBS) with different treatments are shown.

To further verify our findings *in vivo*, the mice that had been inoculated subcutaneously with B16F10 or LL2 cells (10^6^) at day 0 were intraperitoneal injected with PG2 (50 mg/kg) at day 7, day 10, day 13 followed by cisplatin (2 mg/kg) at days 8, 11, and 14, or with either treatment alone. The control mice were treated with PBS. To analyze the safety of the combination therapy, the body weight of mice was measured. As shown in Fig. 5 A and B, the body weight of tumor-bearing mice treated with PG2 was not significantly influenced by three cycle treatments. PG2 significantly rescued the loss of body weight of tumor-bearing mice treated with cisplatin (Fig. 5 A and B). Meanwhile, mice treated with cisplatin did not have the decrease in tumor volume, indicating that low-dose cisplatin cannot significantly inhibit tumor growth. The combination therapy significant reduced tumor growth in two tumor models (Fig. 5 C and D). Interesting, in LL2 tumor model, PG2 had the ability to inhibit the tumor growth, but the phenotype was not observed in B16F10 tumor models. The survival of the tumor-bearing mice treated with PG2 combined with cisplatin was significantly enhanced compared with that of the mice treated with PBS in two tumor models (Fig. 5 E and F). Although the survival of LL2-bearing mice treated with PG2 did not prolong in comparison with that of LL2-bearing mice treated with PBS (P>0.05), the LL2 tumor volume of mice treated with PG2 was smaller than that treated with PBS. Taken together, the results demonstrated that the combination therapy inhibited tumor growth and enhanced the survival of tumor-bearing mice.

### Increased infiltrating T cells in the tumors following the combination therapy of PG2 and cisplatin

**Figure 6:**
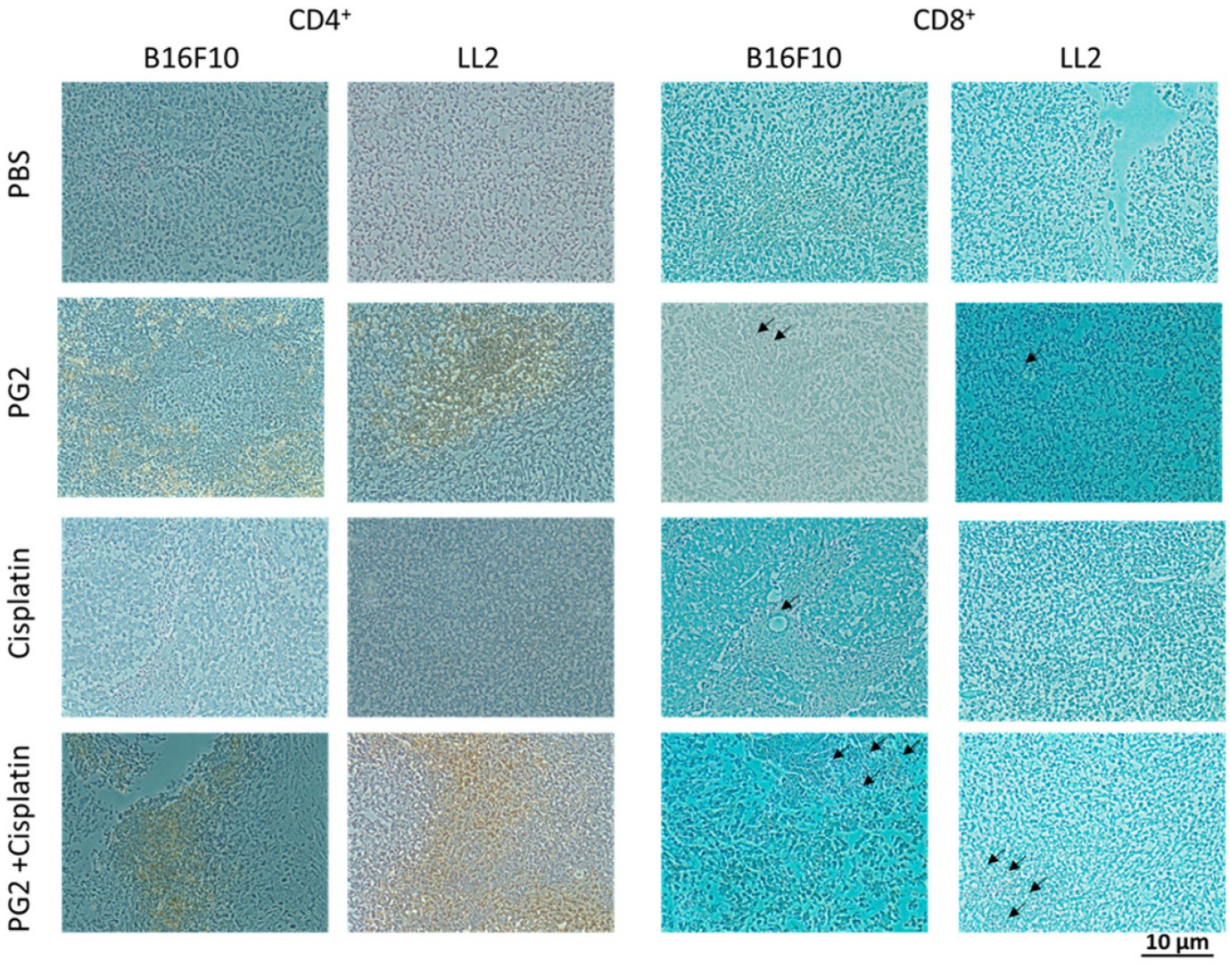
Increases in T-cell infiltrates in the tumors from tumor-bearing mice treated with PG2 in combination with cisplatin. Groups of 4 mice that had been inoculated with B16F10 or LL2 cells (10^6^) at day 0 were intraperitoneal injected with PG2 (50 mg/kg) at day 7, day 10, day 13 followed by cisplatin (2 mg/kg) at days 8, 11, and 14, or with either treatment alone. Vehicle control mice received PBS. The B16F10 or LL2 tumors were excised at day 15 and immunostained with antibodies against CD4^+^ or CD8^+^ (× 200). Arrows indicate the location of positive cells.

The immunohistochemical staining was used to analyze cell infiltrates in tumors from B16F10 or LL2-bearing mice treated with PG2 or cisplatin alone, or in combination. As shown in Fig. 6, CD4^+^ T cells that infiltrated the tumors were observed in the mice treated with PG2 and, in particular, in those treated with PG2 combined with cisplatin. The numbers of infiltrating CD4^+^ T cells in the tumors treated with PG2 plus cisplatin were significantly increased compared with those in the tumors from the remaining three treatment groups, whereas no such difference was found between cisplatin- and PBS-treated groups (Fig. 6). Although there are more infiltrating CD8^+^ T cells in tumor treated with PG2 and PG2 combined with cisplatin group, there were in a very small amount. These results indicate that the combination therapy with PG2 and cisplatin resulted in increasing infiltrating T cells in tumors.

## Discussion

Solid tumors frequently develop multiple drug resistant due to insufficient blood vessels supply. The insufficient blood vessels limit the distribution and activity of chemotherapeutic drugs. Tumor immune escape may be the targets of antitumor therapy 17. Assuming that a drug has dual efficacy is well worth exploring. Herein, we used PG2 as supportive role in chemoresistance, and immune modulation, rather than its direct cytotoxicity on tumor cells. The molecular mechanism that PG2 regulated the expression of Cx43 and IDO was examined. The treatment of PG2 in tumor cells increased Cx43 expression with decreased the protein level of IDO and kynurenine content. The combined therapy significantly inhibited tumor growth and enhanced the survival of tumor-bearing mice. This strategy may be relevant to combined therapy approaches for killing drug-resistant and immune escape population in tumors.

Previously, we found that the expression of Cx43 was reduced after cisplatin treatment 11. A single treatment (cisplatin) cannot induce bystander effect 18. The 5-FU had different effects on the expression of Cx43 (Fig. 3). Type of chemotherapy drug in different tumor cells induced the different effects on connexin protein expression. The molecular mechanism is worth exploring in the future. The PG2 enhanced the sensitivity of the tumor cells to killing by cisplatin that induces cell death. Cx43 could transfer the apoptotic signals, such as cytochrome c, reactive oxygen species, to the neighboring cells and amplify the treatment range 19. The upregulation of Cx43 in tumor cells facilitated antigen transferring between tumor cells and dendritic cells and reduced the tumor immune escape 20. Cx43 is more than a passive channel to provide physical support. It has an active and complex role in regulating the signaling of the cells. Cx43 could reduce tumor growth via a gap junction-independent mechanism 21, 22. The C-terminal region of Cx43 regulates cellular signal transduction and influences the protein expression in cells 23. Recently, Cx43 might be crucial in regulation of tumor-releasing IDO, mediated tumor immune escape 7. Inactivation of Akt in response to PG2 results in the inhibition of IDO, which in turn contributes to the tumor immune escape-associated behaviors. PG2 may involve the tryptophan metabolism enzyme pathway, which is worth further research. Herein, PG2 was significantly reduced the IDO protein expression, which is an intracellular heme-containing enzyme that initiates the first and rate-limiting step of tryptophan degradation.

The activity of PG2 appeared insufficient to inhibit tumor growth or enhance survival of B16F10 tumor-bearing mice. However, PG2 may have pleiotropic activities that can directly and indirectly affect tumor immunity processes in LL2 tumor models. As shown in PG2-treated LL2 tumor model, the slightly growth inhibition of LL2 was observed. The phenomena might be related to PG2-mediated the downregulation of IDO. The tumor growth rate might be critical factor for IDO inhibitor therapy. The growth rate of B16F10 cells was faster than that of LL2 cells *in vivo*. The rapidly growing tumor (B16F10) is more resistant to PG2-mediated active T cell response. The growth rate of tumor might be a critical factor involved PG2-related responses. Meanwhile, the mice treated with PG2 showed a significant reduction in the loss of body weight induced by cisplatin administration, suggesting that PG2 indeed reduced the tumor-related fatigue 5.

Tumors can utilize multiple strategies to evade the immune response, requiring an appropriate method that involves a combination of targets to improve therapeutic outcomes. We explored the potential to enhance the tumor therapeutic response through Cx43 and IDO pathway inhibition in different tumor types including melanoma and lung cancer 25, 26. Recently, we also observed that PG2 could inhibit the expression of programmed cell death protein 1 ligand 1 (PD-L1) in tumor cells (unpublished data). There are many other mechanisms by which the antitumor T cell response is inhibited. Herein, we provided one of possible mechanisms for PG2 antitumor activity. The downregulation IDO or PD-L1 may play a role in reprogramming the tumor microenvironment from an immunosuppressive to an immune permissive environment. The PG2/cisplatin combo therapy may expand the potential of cancer immunotherapy.

## References

[1] Tsao YT (2017). Kuo CY. Kuan YD. Lin HC. Wu LH. Lee CH. The extracts of *Astragalus membranaceus* inhibit melanogenesis through the ERK signaling pathway. Int J Med Sci.

[2] Auyeung KK (2016). Han QB. Ko JK. *Astragalus membranaceus*: A Review of its protection against inflammation and gastrointestinal cancers.

[3] Han R (2016). Tang F. Lu M. Xu C. Hu J. Mei M. Wang H. Protective effects of *Astragalus* polysaccharides against endothelial dysfunction in hypertrophic rats induced by isoproterenol. Int Immunopharmacol.

[4] Leischer T (2016). Chang TT. Chiang JH. Sun MF. Yen HR. Improved survival with integration of Chinese herbal medicine therapy in patients with acute myeloid leukemia: a nationwide population-based cohort study.

[5] Chen HW (2012). Lin IH. Chen YJ et al. A novel infusible botanically-derived drug, PG2, for cancer-related fatigue: a phase II double-blind, randomized placebo-controlled study. Clin Invest Med.

[6] Cheng YJ (2015). Chang MY. Chang WW. Wang WK. Liu CF. Lin ST. Lee CH. Resveratrol enhances chemosensitivity in mouse melanoma model through connexin 43 upregulation. Environ Toxicol.

[7] Lin HC (2017). Yang CJ. Kuan YD. Wang WK. Chang WW. Lee CH. The inhibition of indoleamine 2, 3-dioxygenase 1 by connexin 43. Int J Med Sci.

[8] Trosko JE, Ruch RJ (2002). Gap junctions as targets for cancer chemoprevention and chemotherapy. Curr Drug Targets.

[9] Leone A (2012). Longo C. Trosko JE. The chemopreventive role of dietary phytochemicals through gap junctional intercellular communication. Phytochem Rev.

[10] Wang WK (2015). Chen MC. Leong HF. Kuo YL. Kuo CY. Lee CH. Connexin 43 suppresses tumor angiogenesis by down-regulation of vascular endothelial growth factor via hypoxic-induced factor-1α. Int J Mol Sci.

[11] Chang WW (2013). Lai CH. Chen MC. Liu CF. Kuan YD. Lin ST. Lee CH. *Salmonella* enhance chemosensitivity in tumor through connexin 43 upregulation.

[12] Trosko JE (2007). Gap junctional intercellular communication as a biological "Rosetta stone" in understanding, in a systems biological manner, stem cell behavior, mechanisms of epigenetic toxicology, chemoprevention and chemotherapy. J Membr Biol.

[13] Hou YC (2015). Wu JM. Wang MY. Wu MH. Chen KY. Yeh SL. Lin, M.T. Modulatory effects of *Astragalus polysaccharides* on T-cell polarization in mice with polymicrobial Sepsis. Mediators Inflamm.

[14] Kuan YD (2016). Lee CH. *Salmonella* overcomes tumor immune tolerance by inhibition of tumor indoleamine 2, 3-dioxygenase 1 expression. Oncotarget.

[15] Upham BL, Guzvić M, Scott J, Carbone JM, Blaha L, Coe C, Li LL, Rummel AM, Trosko JE (2007). Inhibition of gap junctional intercellular communication and activation of mitogen-activation protein kinase by tumor-promoting organic peroxides and protection by resveratrol. Nutr Cancer.

[16] Wang WK (2014). Kuan YD. Kuo CY. Lee CH. Connexin 43 gene therapy delivered by polymer-modified Salmonella in murine tumor models. Polymers.

[17] Lee CH (2012). Engineering bacteria toward tumor targeting for cancer treatment: current state and perspectives. Appl Microbiol Biotechnol.

[18] Arora S, Heyza JR, Chalfin EC, Ruch RJ, Patrick SM (2018). Gap junction intercellular communication positively regulates cisplatin toxicity by Inducing DNA damage through bystander signaling. Cancers.

[19] Leone S (2008). Fiore M. Lauro MG. Pino S. Cornetta T. Cozzi R. Resveratrol and X rays affect gap junction intercellular communications in human glioblastoma cells. Mol Carcinog.

[20] Saccheri F (2010). Pozzi C. Avogadri F. Barozzi S. Faretta M. Fusi P. Rescigno M. Bacteria-induced gap junctions in tumors favor antigen cross-presentation and antitumor immunity. Sci Transl Med.

[21] Jiang JX (2005). Gu S. Gap junction- and hemichannel-independent actions of connexins. Biochim Biophys Acta.

[22] de Feijter AW, Matesic DF, Ruch RJ, Guan X, Chang CC, Trosko JE (1996). Localization and function of the connexin 43 gap-junction protein in normal and various oncogene-expressing rat liver epithelial cells. Mol Carcinog.

[23] Giepmans BN, Hengeveld T, Postma FR, Moolenaar WH (2001). Interaction of c-Src with gap junction protein connexin-43: Role in the regulation of cell-cell communication. J Biol Chem.

[24] Zhou R, Chen H, Chen J, Chen X, Wen Y, Xu L (2018). Extract from Astragalus membranaceus inhibit breast cancer cells proliferation via PI3K/AKT/mTOR signaling pathway. BMC Complement Altern Med.

[25] Yang CJ, Kuo CT, Wu LH, Chen MC, Pangilinan CR, Phacharapiyangkul N, Liu W, Chen YH, Lee CH (2019). Eicosapentaenoic acids enhance chemosensitivity through connexin 43 upregulation in murine melanoma models. Int J Med Sci.

[26] Wang CC, Yang CJ, Wu LH, Lin HC, Wen ZH, Lee CH (2018). Eicosapentaenoic acid reduces indoleamine 2,3-dioxygenase 1 expression in tumor cells. Int J Med Sci.

